# Feasibility of identifying plasma donors with high measles neutralizing antibody concentrations for the use of producing a measles hyperimmune globulin for postexposure prophylaxis

**DOI:** 10.1007/s12026-022-09274-z

**Published:** 2022-03-09

**Authors:** Cornelia Lackner, Michael Karbiener, Lukas Faltner, Maria R. Farcet, Thomas R. Kreil

**Affiliations:** Global Pathogen Safety, Takeda Manufacturing Austria AG, Takeda, Benatzkygasse 2-6, 1221 Vienna, Austria

**Keywords:** Plasma, Treatment, Measles virus antibody potency, Intravenous immunoglobulin, Polyclonal neutralizing antibodies, Immunodeficiency

## Abstract

Immune globulin (IG) is administered as measles postexposure prophylaxis (PEP) in people with primary immunodeficiency disorders or individuals not eligible for live virus vaccination. However, measles virus (MeV) neutralizing antibody (nAb) levels in plasma for fractionation and IG products fractionated thereof have declined. Here, the feasibility of producing a measles hyperimmune globulin (HIG) for PEP of high-risk individuals was investigated. Plasma samples (*n* = 384) were selected based on donor self-identification for previous MeV infection or vaccination, to determine the MeV-nAb content and compare it to the potency of plasma pools (*n* = 13) from the current IG manufacture. Convalescent donors have higher mean MeV-nAb concentrations (3.9 IU/mL) than vaccinated donors (2.5 IU/mL), as previously reported. However, their selection would only result in a 1.4-fold elevated nAb concentration compared to current plasma pools, which is not sufficient for HIG production. Interestingly, thirty-two donors (8%) had a MeV-nAb concentration of ≥ 8 IU/mL. The selective use of these plasma donations would result in sixfold higher plasma pool concentrations, which should permit the manufacture of the measles HIG. Further, the longitudinal analysis of a subset of individuals who repeatedly donated plasma at a high frequency revealed only a minor decline (~ 30%) of MeV-nAb levels. Repeat donations of such high-potency donors would thus facilitate the production of the measles HIG. Due to its markedly raised MeV-nAb concentration compared to standard IG, such preparation could significantly shorten infusion time and thus improve the treatment experience for both physicians and patients, especially infants.

## Background

Measles is a highly contagious viral infection. Upon exposure, up to 90% of naive individuals become infected [1]. Despite the availability of safe and effective vaccines, measles still poses a threat to human health. Between 2016 and 2019, measles cases increased > 500% worldwide [2, 3] as a result of vaccination levels below the 90–95% threshold required for herd immunity [4]. The COVID-19 pandemic has further increased the risk of measles outbreaks due to the disruption and suspension of measles vaccination campaigns in 26 countries worldwide [1]. These circumstances put vulnerable groups at high risk of developing serious diseases, especially people with primary immunodeficiency disorders (PID) or individuals not eligible for live virus vaccination, like infants under 6 months of age, pregnant women, and immunosuppressed patients [5]. After exposure, rapid containment measures to halt measles transmissions and PEP to protect the health of exposed individuals from severe complications are required [6]. Vaccination or immune globulin (IG) administration, shown as effective in preventing measles infection when given shortly after exposure [6], is routinely used for measles PEP [5]. Current treatment recommendations suggest administering IG within 6 days of measles exposure [5, 7], and the protective serum level for immunocompetent individuals is estimated at 120 mIU/mL [8, 9]. There is currently no requirement for IG lots released in Europe to be tested for the measles virus (MeV)-neutralizing antibody (nAb) content, but the use of these for MeV-PEP is possible since the 0.36 × CBER standard Lot 176 MeV-nAb concentration threshold can be added to the product specification [10]. Over the past decades, MeV-nAb levels in plasma for fractionation and IG products fractionated thereof have declined [11, 12]. As IG used for PEP seems to show a nAb concentration-dependent effect, with higher anti-measles concentrations providing superior protection [5, 6], the availability of a high potency IG would be of great clinical benefit. Here, based on donor self-identification for previous MeV infection or vaccination, we examined the feasibility of identifying plasma donors with high measles neutralizing antibody concentrations for producing a measles hyperimmune globulin (HIG), per definition to contain at least five times the antibody potential of the standard preparation [13], for more effective PEP of high-risk groups.

## Methods

### Plasma samples

A total of 384 plasma samples were collected in two BioLife Europe donation centers in Austria from May to September 2020. Samples were grouped according to donor self-identification: either a natural MeV infection (“convalescent”) or receipt of 2 doses of a measles vaccine (“vaccinated”) was requested of the donor, without the requirement of documented proof to participate in the study (Table 1). Donors who claimed to have both gone through infection and vaccination (*n* = 10), were assorted to the “convalescent” group. In order to set the baseline for calculating the minimum nAb concentration of a plasma pool needed for HIG production, 10 plasma pool samples (each pooled in 2020 from 96 Austrian donations, Takeda Plasma Analytics) and 3 plasma pool samples derived from IG production (each a pool of several thousands of plasma donations collected in the EU, Takeda Vienna production site) were tested for MeV-nAbs. Plasma samples for the characterization of MeV-nAb concentrations in high-frequency donors originated from BioLife US plasma donation centers. One hundred thirty-six samples of 9 donors, designated as high-frequency donors in this study, with an average of 15 donations during a time period of fewer than 3 months were tested. All donors signed informed consent.

**Table 1:** Information on donors, samples, and the summary of neutralization assay results

	Convalescent	Vaccinated	Pools
Samples-male	118	72	n.a
Samples-female	69	90	n.a
Samples-gender not detailed	18	11	n.a
Samples below LOD	4	2	n.a
Total number of samples	209	175	13
Mean age (years)	42	34	n.a
Mean MeV-nAb concentration (IU/mL)	3.9 ± 0.4	2.5 ± 0.3	2.7 ± 0.1
Mean MeV-nAb concentration (IU/mL)-male	3.3 ± 0.5	2.7 ± 0.6	n.a
Mean MeV-nAb concentration (IU/mL)-female	4.8 ± 0.8	2.4 ± 0.3	n.a

LOD: < 0.2–0.7 IU/mL; nAb concentrations are expressed as arithmetic mean ± standard error of the mean (SEM)

### Detection of measles virus-neutralizing antibodies

MeV-nAb concentrations were determined in duplicates for donor plasma and plasma pool samples, except for samples originating from high-frequency donors that were tested in single determinations. First, samples were diluted 1:20 in cell culture medium and then serially diluted in twofold steps and mixed 1:2 with the infectious measles virus (strain Edmonston), adjusted to 10^3^ tissue culture infectious dose per milliliter. After 150 min incubation at room temperature, the sample/virus mix was plated on Vero cells in eight repeats per dilution, seeded in 96-well plates (2 × 10^5^/mL, 100 µL/well) on the day of infection. After a 7-day incubation period, cells were microscopically evaluated for the appearance of a cytopathic effect. The MeV-nAb titer (i.e., the reciprocal sample dilution resulting in 50% virus neutralization) was determined using the Spearman–Kaerber formula. To allow for data comparison between different test days, sample nAb concentrations were normalized against an internal standard (plasma) run within every assay. This internal standard was calibrated in a series of independent assays against the WHO reference standard (National Institute for Biological Standards and Control code 97/648) to determine a conversion factor between these two standards. For the samples analyzed in a particular assay, the anti-MeV concentration of the internal standard analyzed in the same assay, together with the conversion factor, enabled the conversion of antibody concentrations to IU/mL. The neutralization assay included several validity criteria to monitor assay performance and allow for data comparison between different assays. These criteria included confirmation of virus infectivity before and after the incubation period, cell viability, and a neutralization control, which was tested in duplicate in every assay to check for assay validity (anti-measles potency within the range of − 3 × SD to + 3 × SD). Based on repeat testing of the neutralization control, the assay’s coefficient of variation was calculated as 28%. The LOD was defined as below the result of a single well with neutralization (i.e., lack of cytopathic effect) and depended on the neutralization titer of the internal standard on the particular day. Throughout the study, the LOD was < 0.2–0.7 IU/mL. In the entire manuscript, descriptive statistics are given as arithmetic mean ± standard error of the mean (SEM).

## Determination of anti-measles potency threshold for the HIG plasma selection

Starting at a cutoff of 4 IU/mL, the plasma donation samples with a measles antibody concentration above that cutoff were selected, and an average measles antibody concentration for this subgroup was calculated. The value was divided by the average measles antibody concentration of the 13 analyzed plasma pool samples (i.e., 2.7 IU/mL) to obtain the fold enrichment. The calculation was repeated in increments of 1 IU/mL up until 14 IU/mL. By this, a cutoff of 8 IU/mL was identified as the first value leading to a ≥ sixfold enrichment.

### Graphs and statistical analysis

MeV-nAb concentrations were calculated using Microsoft Excel 365 Pro Plus (version 2008). Graphical illustrations and statistical analysis (unpaired *t* test) were done using Graph Pad Prism v8.1.1 software.

## Results

Plasma donations of 384 study participants who self-identified either for prior MeV infection or full measles vaccination were screened for MeV-nAb concentrations. 54% and 46% of samples obtained from these donors fell within the convalescent and vaccinated groups, respectively (Table 1). The mean age of 42 years for the convalescent group was significantly higher (*p* < 0.0001) than the mean age of 34 years for the vaccinated group. Six donations had MeV-nAb concentrations below the limit of detection (LOD) and were excluded from further calculations. On average, convalescent donors showed 1.6-fold higher nAb concentrations than vaccinated persons (3.9 ± 0.4 IU/mL vs. 2.5 ± 0.3 IU/mL, *p* = 0.004, unpaired *t* test, Table 1). No significant differences in MeV-nAb levels were found within the convalescent and the vaccinated group with respect to gender (*p* > 0.05, Table 1).

**Fig. 1:**
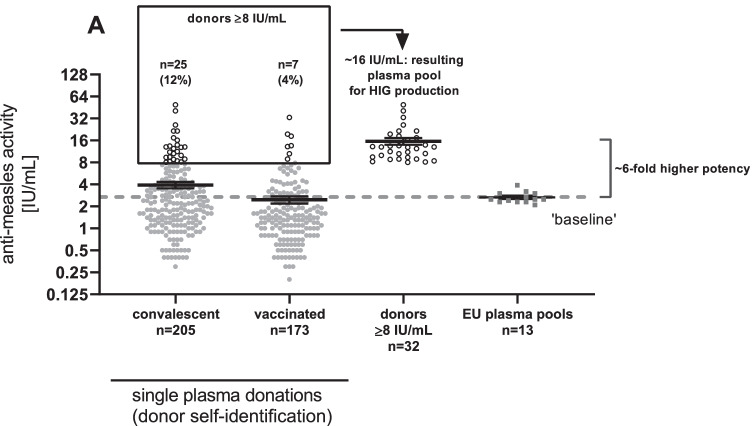
Measles virus-neutralizing antibody concentrations in convalescent and vaccinated individuals and EU plasma pools. Anti-measles activity [IU/mL] of samples is shown as dots (log_2_ scale) overlaid by the arithmetic mean ± SEM. Donations above the threshold of 8 IU/mL are depicted as open dots. The dashed line represents the “baseline” at 2.7 IU/mL, set by the arithmetic mean of the pooled plasma donation group

Pooled plasma samples, used for setting the baseline of the minimum MeV-nAb plasma pool concentration needed for HIG production, showed a mean MeV-nAb concentration of 2.7 ± 0.1 IU/mL (Table 1). In comparison, the mean nAb concentration determined for the convalescent group (3.9 ± 0.4 IU/mL) was 1.4-fold above that baseline. As a fivefold increased nAb concentration compared to the antibody potency of a standard preparation is expected for HIG production [13], the 1.4-fold increase achievable when plasma from donors self-identified for prior MeV infection is selected would not be sufficient for HIG production (Fig. 1).

In order to obtain high-potency starting material with at least fivefold higher MeV-nAb content for HIG production, the possibility of selecting donations with MeV-nAb concentrations above a certain threshold was evaluated. A threshold of 8 IU/mL, which should result in a sixfold increase in potential plasma pool potency in relation to the baseline, was explored with the donor population studied. In the current study population, 25 donations (12%) from convalescent donors and 7 donations (4%) from vaccinated donors met this criterion, and if donations of these donors were to be pooled, a plasma pool for HIG production with a potency of 16 IU/mL could be expected (Fig. 1).

**Fig. 2:**
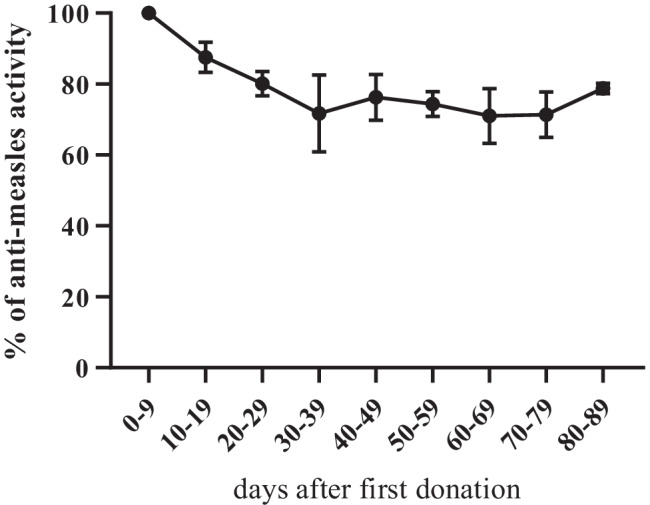
Longitudinal analysis of the measles virus-neutralizing antibody levels of nine high-frequency donors. Days after first plasma donation (bins of ≤ 10 days), plotted against the percentage of anti-measles activity in relation to the first bin value (0–9 days after the first donation). Each dot depicts the mean value of 9 donors per bin with SEM error bars (up to 4 plasma donations per donor and bin)

Due to the small number of high-potency donors, repeat donations of these would be needed to support HIG production. To investigate the robustness of MeV-nAb levels in a setting of prolonged periodic plasma donation, samples of 9 high-frequency donors were assessed (Fig. 2). The number of donations per individual ranged from 10 to 19 within a period of approximately 3 months. Only a minor decay of MeV-nAb activity in these high-frequency donors was seen, to a maximum of 30% after a mean of 15 donations (Fig. 2).

## Discussion

The measured MeV-nAb concentration ratio of 1.6 between vaccinated and convalescent donors confirmed the trend of higher nAb concentration levels in donors with a history of natural infection [4]. However, the difference between the two study groups was smaller than expected, likely due to donor self-identification rather than documented evidence of convalescent vs. vaccinated individuals. The two study groups differed significantly in terms of mean age, as people that had undergone measles are more likely to be born before the onset of vaccination campaigns, 1974 in Austria [14]. A comparable study conducted in 2020 reported a ratio of 3.3 between vaccinated and naturally infected persons in Italy [4], which indicates that the use of vaccination records as carried out by Anchini et al. [4] is more precise to discriminate individuals with respect to their vaccination or infection history. Still, donor selection on this approach would not allow for meeting the WHO-defined fivefold enrichment target for HIG classification [13]. When comparing the MeV-nAb concentrations of convalescents determined in this study to the baseline of 2.7 IU/mL, which represents the expected potency of starting material for current standard IGs, the enrichment factor was only 1.4. When donors were grouped in bins of ≤ 5 years according to their birth date, a decrease of MeV-nAb concentrations was seen for donors born after 1979 (data not shown), shortly after the first recommendation to vaccinate against MeV in Austria in 1974 [14]. This is in line with Bechini et al. [15], who showed MeV-nAb concentrations to significantly increase with age, due to the combined effect of natural infection and active immunization. However, for our donor collective, even when selecting the bin showing the highest overall anti-measles activity (birth years 1965–1969, convalescent), only a 2.3-fold enrichment of MeV-nAbs versus the baseline could be achieved. These data indicate that also donor selection according to age is not sufficient for HIG starting material selection.

Alternatively, the selection of donors with MeV-nAb concentrations ≥ 8 IU/mL was identified as a viable approach for the selection of plasma for HIG production. This results in a plasma pool of ~ 16 IU/mL and a sixfold increase compared to the baseline of 2.7 IU/mL. The safety margin of a sixfold increase rather than a fivefold enrichment would ensure the necessary MeV-nAb levels even with probable fluctuations in donor population or plasma pooling.

In this study, only 8% of the participating plasma donors showed MeV-nAb concentrations above the threshold of 8 IU/mL. Thus, repeat donations of these donors would be desirable to allow for efficient HIG production. Indeed, this is possible, as longitudinal analysis of MeV-nAb levels in the regular donor population revealed only a slight decline of ~ 30% antibody potency even during intensive donor plasmapheresis (Fig. 2). Calculating a safety margin of a sixfold increase also partially covers a possible decline of antibody concentrations in donors undergoing repeated plasmapheresis. Additional pretesting of donor mini pools prior to manufacturing, or the manufacturing starting pool, could also ensure appropriate plasma pooling in order to provide high potency starting material for HIG production.

High coverage with 2 doses of measles, mumps, and rubella (MMR) vaccine in the population as a preventive measure remains the optimal way to protect individuals from measles [16]. However, in terms of decreased vaccination coverage, which has been additionally aggravated by the current COVID-19 pandemic [17], vaccination and IG PEP are of great importance and recommended for naive persons exposed to measles and individuals most at risk for severe disease and complications, e. g., immunosuppressed individuals or pregnant women, respectively [7].

Even though standard IG can be used for measles PEP [10], studies indicate higher effectiveness of timely given high-dosage IG [6] or high-potency IG administered intramuscularly [18], indicating a dose–response effect of immunoglobulin in PEP against measles. Thus, the preselection of high-potency starting material and repeat donations of these donors for HIG production seems desirable, even more as MeV-nAb concentrations in IGs have decreased in the past [11, 19]. For HIG manufacture, the WHO has suggested a minimum enrichment of fivefold over a regular IG [13]. To further raise the anti-measles potency to a tenfold enrichment, a 20% subcutaneous HIG might be used. Assuming a linear enrichment of measles neutralizing antibodies from a 16-U/mL starting plasma pool to a 20% subcutaneous HIG, such a product would exceed the minimum potency that is required by the US Food and Drug Administration (FDA) by a factor of about 22-fold [20]. Therefore, a subcutaneous 20% HIG would allow for administration of higher doses of nAbs, a marked reduction of treatment time, greater ease of administration, and shortened hospital stay, important factors when children or infants need to be treated. Overall, a subcutaneously administered anti-measles HIG in those who are nonimmune, have been exposed, and are most at risk of severe disease and complications could enable a convenient (for both, patients and physicians) and well-tolerated form of disease prevention, especially for younger children and infants.

## Data Availability

Not applicable.
